# Genotyping and Zoonotic Potential of *Enterocytozoon bieneusi* in Pigs in Xinjiang, China

**DOI:** 10.3389/fmicb.2019.02401

**Published:** 2019-10-22

**Authors:** Dong-Fang Li, Ying Zhang, Yu-Xi Jiang, Jin-Ming Xing, Da-Yong Tao, Ai-Yun Zhao, Zhao-Hui Cui, Bo Jing, Meng Qi, Long-Xian Zhang

**Affiliations:** ^1^College of Animal Science, Tarim University, Alar, China; ^2^College of Animal Science and Veterinary Medicine, Henan Agricultural University, Zhengzhou, China

**Keywords:** *Enterocytozoon bieneusi*, infection rate, novel genotype, potential zoonotic, pig

## Abstract

*Enterocytozoon bieneusi* is an obligate intracellular fungus, infecting various invertebrate and vertebrate hosts, it is common in humans and causes diarrhea in the immunocompromised. In the present study, 801 fecal specimens were collected from pigs on seven large-scale pig farms in Xinjiang, China. Nested polymerase chain reaction (PCR) amplification of the internal transcribed spacer (ITS) gene showed that the overall *E. bieneus*i infection rate was 48.6% (389/801). The *E. bieneusi* infection rates differed significantly among the collection sites (20.0–73.0%) (χ^2^ = 75.720, *df* = 6, *p* < 0.01). Post-weaned pigs had the highest infection rate (77.2%, 217/281), followed by fattening pigs (67.4%, 87/129) and pre-weaned suckling pigs (35.5%, 60/169). Adult pigs had the lowest infection rate (11.3%, 25/222). The *E. bieneusi* infection rates also differed significantly among age groups (χ^2^ = 246.015, *df* = 3, *p* < 0.01). Fifteen genotypes were identified, including 13 known genotypes (CHC, CS-1, CS-4, CS-7, CS-9, D, EbpA, EbpC, EbpD, H, PigEb4, PigEBITS5, and WildBoar8) and two novel genotypes (XJP-II and XJP-III). Among them, six genotypes (CS-4, D, EbpA, EbpC, H, and PigEBITS5) have been reported in humans. Phylogenetic analysis showed that all the genotypes belonged to Group 1 of *E. bieneusi*. These findings suggest that pigs may play an important role in transmitting *E. bieneusi* infections to humans.

## Introduction

Over 1300 microsporidial species infect a variety of invertebrate and vertebrate hosts, *Enterocytozoon bieneusi* is considered the most common microsporidial species to cause opportunistic infections in humans (Sak et al., 2011; Matos et al., 2012). *E. bieneusi* infections cause diarrhea, malabsorption and possible lung pathologies, and host health status is the main influencing factor (del Aguila et al., 1997; Matos et al., 2012). *E. bieneusi* is transmitted mainly via the fecal-oral route through ingestion of contaminated water or food or accidental ingestion of spores eliminated in the feces of infected animals or humans (Stentiford et al., 2016).

Analysis of the ribosomal internal transcribed spacer (ITS) nucleotide sequence polymorphism is widely used for *E. bieneusi* molecular typing (Santín and Fayer, 2011; Zhang et al., 2011; Karim et al., 2014; Liu et al., 2017; Deng et al., 2018). Over 470 *E. bieneusi* genotypes have been identified from the ITS gene in humans, mammals, birds and water; however, this pathogen also exists in many undefined areas worldwide (Henriques-Gil et al., 2010; Li D. et al., 2019). Phylogenetic analysis has revealed high diversity and genetic variation among isolates from human and animal origins, and these isolates are clustered into 11 major genetic groups (Galván-Díaz et al., 2014; Wang et al., 2019).

Studies have identified zoonotic *E. bieneusi* genotypes from pigs in China, thus implicating pigs as dispersing agents and a potential source of human infections (Li et al., 2014a,b,^2017^; Li W. et al., 2019; Zhao et al., 2014; Wan et al., 2016; Wang et al., 2018a; Zou et al., 2018; Luo et al., 2019). In China, Xinjiang Uygur Autonomous Region (hereafter referred to as Xinjiang) lies in inland Eurasia and has a typical half-arid/arid climate (34°25′–48°10′ N, 73°40′–96°18′ E). It is the largest provincial-level administrative region by land area and a historically important passage of the ancient silk road. Information on the occurrence of *E. bieneusi* in pigs in Xinjiang is scarce; therefore, this study was conducted to examine the occurrence of *E. bieneusi* in pigs in Xinjiang, China, and to assess the zoonotic transmission risk of this pathogen.

## Materials and Methods

### Ethics Statement

Permission was obtained from animal owners or managers before collecting specimens, and no specific permits were required for the described field studies. All work involving animals was carried out in accordance with the Regulations for the Administration of Affairs Concerning Experimental Animals. The Research Ethics Committee of Henan Agricultural University reviewed and approved our study (approval no. LVRIAEC 2017-019).

### Fecal Specimen Collection, DNA Extraction, and Purification

Eight hundred one fresh fecal specimens were collected from Duroc and Landrace pigs on seven large-scale intensive pig farms in Xinjiang between September 2017 and June 2018. Each sampled farm contained 10000–80000 pigs. All farms were visited on a single occasion. A veterinarian randomly collected the fecal specimens either from the rectum or from the internal portion of a stool sample on the ground avoid possible contamination from the specimen surface touched the ground. All specimens (approximately 5–30 g) were collected using sterile disposal latex gloves; marked with the date, age, and farm; stored in insulated boxes; and transferred to the laboratory. Collected specimens included 169 fecal specimens from pre-weaned suckling pigs (<20 days old), 281 specimens from post-weaned suckling pigs (21–70 days old), 129 specimens from fattening pigs (71–180 days old), and 222 specimens from sows (>181 days old).

Genomic DNA was directly extracted from the fecal specimens (approximately 200 mg) using the E.Z.N.A.^®^ Stool DNA Kit (Omega Biotek Inc., Norcross, GA, United States) per the manufacturer’s instructions with minor modifications. The extracted DNA was stored at −20°C prior to polymerase chain reaction (PCR) analysis.

### PCR Amplification and Sequence Analysis

*Enterocytozoon bieneusi* was identified via nested PCR amplification and sequencing of the ITS region of the rRNA gene. The primers and thermal cycle parameters used for the two PCR amplifications have been described previously (Buckholt et al., 2002). The outer primers were EBITS3 (5′-GGTCATAGGGATGAAGAG) and EBITS4 (5′-TTCGAGTTCTTTCGCGCTC), and the cycling parameters were 35 cycles of 94°C for 30 s, 57°C for 30 s, and 72°C for 40 s. The inner primers were EBITS1 (5′-GCTCTGAATATCTATGGCT) and EBITS2.4 (5′-ATCGCCGACGGATCCAAGTG), and the cycling parameters were 30 cycles of 94°C for 30 s, 55°C for 30 s, and 72°C for 40 s. The 2×EasyTaq PCR SuperMix (TransGene Biotech Co., Beijing, China) was used for PCR amplification. All PCR assays included both a positive control (DNA from dairy cattle-derived genotype I) and a negative control (distilled water). PCR amplification was repeated twice for each specimen.

Positive secondary PCR products (∼390 bp) were sequenced by GENEWIZ (Suzhou, China), and all products were sequenced in both directions to ensure accurate sequencing results. ClustalX 2.1^1^ was used to align the resulting DNA sequences. Sequences obtained were aligned with reference sequences downloaded from the National Center for Biotechnology Information^2^ to determine genotypes. The nucleotide sequences obtained in the present study were submitted to GenBank^3^ under accession numbers MK778892–K778899 and MK778901–MK778907.

### Phylogenetic and Statistical Analysis

Bayesian inference (BI) and Monte Carlo Markov chain methods were used to construct phylogenetic trees in MrBayes, version 3.2.6^4^. The posterior probability values were calculated by running 1,000,000 generations. A 50% majority-rule consensus tree was constructed from the final 75% of the trees generated via BI. Analyses were run three times to ensure convergence and insensitivity to priors.

The Statistical Package for the Social Sciences (SPSS, version 22.0, available at https://www.ibm.com) was used for the statistical analyses, including Fisher’s exact test and 95% confidence intervals. Differences with *p* < 0.05 were considered significant.

## Results and Discussion

In the present study, the overall *E. bieneusi* infection rate in pigs was 48.6% (389/801) (Table 1), which was higher than most previously reported rates from Chinese provinces, including Guangdong (26.4%, 19/72) (Zou et al., 2018), Inner Mongolia (37.5%, 3/8) (Li et al., 2014a,c), Jilin (43.9%, 145/330) (Zhang et al., 2011; Li et al., 2014a,b,c; Wan et al., 2016), Liaoning (17.4%, 13/73) (Li et al., 2014c; Wan et al., 2016), Sichuan (36.9%, 230/623) (Li et al., 2017; Luo et al., 2019), Tibet (43.2%, 309/715) (Li W. et al., 2019), Yunnan (29.5%, 59/200) and Zhejiang (37.9%, 47/124) (Zou et al., 2018), but lower than that from Shaanxi (78.9%, 442/560) (Wang et al., 2018b; Table 2). The differences in *E. bieneusi* infection rates may be partially attributed to differences in feeding densities, comparisons with cages that had lower pig densities, and greater opportunities for *E. bieneusi* transmission among animals in high densely packed cages (Wang et al., 2018b).

**TABLE 1:** *Enterocytozoon bieneusi* occurrence and genotype distribution in pigs in Xinjiang, China.

**Collection site**	**No. positive/No. specimens**	**% (95 CI)**	***Enterocytozoon bieneusi* genotypes (n)**
Marabishi	48/98	49.0 (38.9–59.1)	CHC5 (1), CS-7 (3), **D** (6), **EbpA** (1), ***EbpC*** (34), EbpD (1), **PigEBITS5** (2),
Alaer	19/95	20.0 (11.8–28.2)	**D** (1), ***EbpC*** (17), **H** (1)
Yarkant	63/130	48.5 (39.8–57.2)	CHC5 (1), **D** (8), ***EbpA*** (43), **EbpC** (9), **PigEBITS5** (2),
Baicheng	67/99	67.7 (58.3–77.1)	***EbpA*** (50), **EbpC** (10), **PigEBITS5** (7)
Shaya	73/100	73.0 (64.1–81.9)	CS-1 (3), CS-4 (20), CS-9 (1), ***EbpA*** (11), ***EbpC*** (11), EbpD (3), PigEb4 (12), **PigEBITS5** (6), WildBoar8 (3), XJP-II (2), XJP-III (1)
Changji	49/130	37.7 (29.3–46.1)	**EbpA** (19), ***EbpC*** (27), EbpD (1), **PigEBITS5** (2)
Ruoqiang	70/149	47.0 (38.9–55.1)	CS-1 (2), **D** (2), **EbpA** (5), ***EbpC*** *(60)*, **H** (1)
Total	389/801	48.6 (45.1–52.0)	CHC5 (2), CS-1 (5), **CS-4 (20)**, CS-7 (3), CS-9 (1), **D (17)**, **EbpA (129)**, ***EbpC (168)***, EbpD (5), **H (2)**, PigEb4 (12), **PigEBITS5 (19)**, WildBoar8 (3), XJP-II (2), XJP-III (1)

*Genotypes detected in humans are in bold, and dominant genotypes are in italics.*

**TABLE 2:** *Enterocytozoon bieneusi* occurrence and genotype in pigs in China.

**Province**	**No. of positive/No. of examined (%)**	***Enterocytozoon bieneusi* genotypes (n)**	**References**
Guangdong	19/72(26.4%)	EbpA*^a^* (1), ***EbpC*** (17), GD1 (1)	Zou et al., 2018
Heilongjiang^c^	351/641(54.8%)	CC-1 (2), CHN7/O (1), CS-1 (8), CS-1/EbpC (1), CS-2 (1), CS-3 (1), CS-3/EbpA*^a^* (2), CS-4 (34), CS-5 (1), CS-6 (1), CS-7 (1), CS-8 (4), CS-10 (1), D (20), EbpA*^a^* (37), EbpA*^a^*/EbpC (4), EbpA*^a^*/Henan-IV (1), EbpB (28), EbpB/EbpC (1), ***EbpC*** (61), EbpC/Henan-IV (1), EbpC/O (30), EbpD (1), H (18), Henan-IV (6), HLJ-I (2), HLJ-II (1), HLJ-III (1), HLJ-IV (1), LW1 (1), O (18), PigEBITS5*^a^*/Henan-IV (1)	Li et al., 2014a,c; Zhao et al., 2014; Wan et al., 2016
Henan^b^	744/1372(54.2%)	CHC5 (4), CM8 (11), ***EbpA****^a^* (154), ***EbpC*** (278), G (10), H (14), HN-1 (6), HN-2 (2), HN-3 (1), HN-4 (1), Henan-III (1), LW1 (12), PigEBITS4*^a^* (24), PigEbITS5*^a^* (17), XZP-II (1)	Wang et al., 2018a; Li W. et al., 2019
Inner mongolia	3/8(37.5%)	CHN7 (1), EbpC (1), O (1)	Li et al., 2014a,c
Jilin^c^	145/330(43.9%)	CHN1 (4), CHN7 (11), CHN8 (1), CHN9 (1), CHN10 (2), CS-1 (3), CS-1/G (1), CS-4 (4), CS-6/EbpA*^a^* (1), CS-8 (1), CS-9 (1), CS-9/EbpB (6), CS-9/EbpD (1), EBITS3 (1), ***EbpA***^a^ (34), EbpA*^a^*/EbpC (8), ***EbpC*** (30), H/EbpC (1), Henan-III (1), Henan-IV (2), LW1^a^ (4), O (2)	Li et al., 2014a,b,c; Wan et al., 2016; Zhang et al., 2011
Liaoning^c^	13/73(17.4%)	EbpB/EbpC (6)	Li et al., 2014c; Wan et al., 2016
Shaanxi	442/560(78.9%)	CHC5 (31), CHG3 (1), CHN7 (1), CS-4 (1), D (1), EbpA*^a^* (20), H (4), Henan-IV (3), PigEB4 (3), PigEBITS4*^a^* (33), PigEBITS5*^a^* (13), SHZA1 (2), SHZC1 (1), SLTC1 (2), ***SLTC2*** (59), SLTC3 (15), SMXB1 (1), SMXC1 (1), SMXD1 (1), SMXD2 (1), SYLA1 (2), SYLA2 (1), SYLA3 (1), SYLA4 (1), ***SYLA5*** (56), SYLC1 (1), SYLD1 (1), SZZA1 (1), SZZA2 (8), SZZB1 (1), SZZC1 (3), ***SZZD1*** (81), SZZD2 (1)	Wang et al., 2018b
Sichuan	230/623(36.9%)	CHC5 (10), D (1), EbpA*^a^* (22), ***EbpC*** (143), Henan-IV (24), PigEBITS4*^a^* (12), PigEBITS5 (1), SH8*^a^* (6), WildBoar 7 (1), WildBoar 8*^a^* (7), WildBoar 11 (1), SC02 (1), SCT01 (1), SCT02 (1)	Li et al., 2017; Luo et al., 2019
Tibet	309/715(43.2%)	D (1), ***EbpC*** (302), I (2), XZP-I (1), XZP-II (3)	Li W. et al., 2019
Yunnan	59/200(29.5%)	D (1), EbpA*^a^* (15), ***EbpC*** (31), G (1), H (1), Henan-IV (6), PigEBITS5*^a^* (1), YN1 (1), YN2 (2), YN3 (1)	Zou et al., 2018
Zhejiang	47/124(37.9%)	CAF-1 (2), EbpA*^a^* (2), ***EbpC*** (39), PigEBITS5*^a^* (2), ZJ1 (1), ZJ2 (1)	Zou et al., 2018

**^*a*^Enterocytozoon bieneusi* genotypes with synonyms were amended using the nomenclature system established by Santín and Fayer, 2009: EbpA (synonyms: CHS5, F), PigEBITS4 (synonyms: CHG19, PEbD), PigEBITS5 (synonyms: PEbA), SH8 (synonyms: WildBoar 10), and WildBoar8 (synonyms: WildBoar9). *^*b*^*Partial PCR samples were selected for sequencing. *^*c*^*Partial PCR samples were not successfully sequenced. Dominant genotypes are in italics.*

The *E. bieneusi* infection rates significantly differed among the collection sites (χ^2^ = 75.720, *df* = 6, *p* < 0.01). The highest infection rate was found on a farm from Shaya (73.0%, 73/100) (Table 1). The *E. bieneusi* infection rate also differed significantly among age groups (χ^2^ = 246.015, *df* = 3, *p* < 0.01). Post-weaned pigs had the highest infection rate (77.2%, 217/281), while adult pigs (sows) had the lowest infection rate (11.3%, 25/222) (Table 3). Most previous studies included information on *E. bieneusi* for different age groups of pigs in China. High *E. bieneusi* infection rates were found in pre-weaned and post-weaned pigs from Heilongjiang (78.4%, 87/111; 70.4%, 50/71), Jilin (68.8%, 22/32; 74.6%, 53/71), Zhejiang (54.7%, 76/139), and Henan Provinces (54.2%, 137/253) (Li et al., 2014b,Li W. et al., 2019; Wan et al., 2016; Zou et al., 2018). The higher prevalence among post-weaned pigs may have been due to lower immunity and stress from early wean (Wang et al., 2018b). However, high *E. bieneusi* infection rates were found in fattening pigs from Liaoning (100%, 3/3), Jilin (35.7%, 20/56), Tibet (75.5%, 173/229) and Yunnan Provinces (21%, 42/200) (Wan et al., 2016; Zou et al., 2018; Li W. et al., 2019). The differences in *E. bieneusi* infection rates in these age groups may be partially attributed to differences in geoecology, rearing conditions, animal husbandry, and feeding densities.

**TABLE 3:** *Enterocytozoon bieneusi* occurrence and genotypes in pigs of different ages.

**Age (days)**	**No. positive/No. specimens**	**% (95 CI)**	***Enterocytozoon bieneusi* genotypes (n)**
Pre-weaned	60/169	35.5 (28.2–42.8)	***EbpC*** (24), **EbpA** (12), **D** (11), PigEb4 (5), **PigEBITS5** (4), **CS-4** (3), WildBoar8 (1)
Post-weaned	217/281	77.2 (72.3–82.2)	***EbpC*** (105), **EbpA** (64), **PigEBITS5** (11), CS-4 (9), **D** (6), PigEb4 (5), EbpD (4), CS-1 (4), CS-7 (3), WildBoar8 (2), CHC5 (1), **H** (1), XJP-1 (2)
Fattening pigs	87/129	67.4 (59.2–75.6)	***EbpA*** (46), **EbpC** (26), **CS-4** (7), **PigEBITS5** (3), CHC5 (1), CS-1 (1), CS-9 (1), **H** (1), XJP-2 (1)
Sow	25/222	11.3 (7.1–15.5)	***EbpC*** (13), **EbpA** (7), PigEb4 (2), **CS-4** (1), EbpD (1), **PigEBITS5** (1)

*Genotypes detected in humans are in bold and dominant genotypes were in italics.*

From 389 positive specimens, 15 *E. bieneusi* genotypes were identified from nucleotide sequences via ITS-PCR. These included 13 known genotypes (CHC5, CS-1, CS-4, CS-9, CS-7, D, EbpA, EbpC, EbpD, H, PigEb4, PigEBITS5, and WildBoard8) and 2 novel genotypes (named XJP-II and XJP-III; Table 1). EbpC (43.2%, 168/389) and EbpA (33.2%, 129/389) were the predominant genotypes in pigs in Xinjiang (Table 1). The dominant genotypes also varied across sample regions. Genotype EbpC was dominant in Marabishi, Alaer, Changji, and Ruoqiang; genotype EbpA was dominant in Yarkant and Baicheng; and genotype CS-4 was dominant and identified only in Shaya (Table 1). In addition, the *E. bieneusi* genotype distribution differed among ages. Genotype EbpC was predominant in pre-weaned, post-weaned, and adult pigs, while EbpA predominated in fattening pigs. These results were similar to those reported for Guangdong, Henan, Jilin, Sichuan, Tibet, Yunnan and Zhejiang Provinces in China, where reported genotypes EbpC and EbpA were predominant in pigs (Li et al., 2014a,b,^2017^,Li W. et al., 2019; Zhao et al., 2014; Wan et al., 2016; Wang et al., 2018a; Zou et al., 2018; Luo et al., 2019; Table 2).

Of the 120 *E. bieneusi* genotypes reported in pigs worldwide (Sak et al., 2008; Němejc et al., 2014; Fiuza et al., 2015; Prasertbun et al., 2017; Wang et al., 2018a,b), over 80 have been reported in pigs in China (Wan et al., 2016; Wang et al., 2018b,c; Li W. et al., 2019). Fifteen genotypes were identified in the present study, of which, six (CS-4, D, EbpA, EbpC, H and PigEBITS5) have been identified in humans. Genotypes CS-4, D, EbpA, EbpC, and H were identified in children and HIV/AIDS patients from China (Wang et al., 2013; Yang et al., 2014; Liu et al., 2017). These results suggest that pigs play an important role in transmitting *E. bieneusi* to humans and other animals.

**FIGURE 1:**
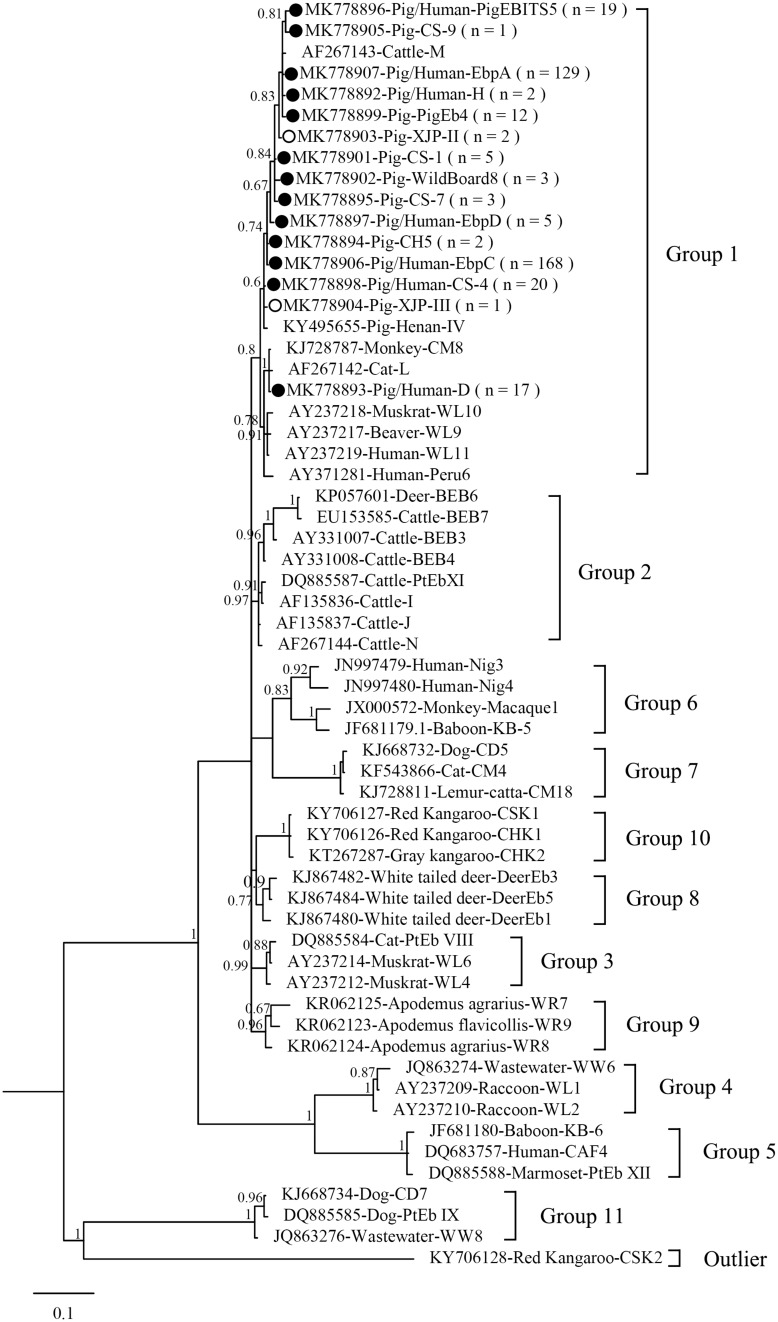
Phylogenetic tree based on Bayesian analysis of the ITS sequences. Statistically significant posterior probabilities are indicated on the branches. Known and novel *Enterocytozoon bieneusi* genotypes identified in the present study are indicated by filled and hollow circles, respectively.

Figure 1 shows the phylogeny of the ITS sequences from the 15 genotypes identified in the present study, and all genotypes identified were classified in Group 1. Accumulating evidence suggests that genotypes in Group 1 have significant zoonotic importance but no strong host specificity (Wan et al., 2016; Li and Xiao, 2019; Li W. et al., 2019). Although direct evidence linking human infections to *E. bieneusi* of animal origin is lacking, direct contact with pigs or with a water supply contaminated by pig waste are considered significant risk factors for zoonotic transmission (Cama et al., 2007).

## Conclusion

This study revealed that *E. bieneusi* is common in pigs in Xinjiang, China. Thirteen known genotypes and two novel genotypes (XIP-II and XIP-III) were classified in Group 1, and showed six of the 15 identified genotypes have been found in humans, indicating that pigs may be reservoirs for zoonotic transmission of human microsporidiosis. These findings extend the knowledge of the *E. bieneusi* distribution among pigs in China.

## Data Availability Statement

The datasets generated for this study can be found in GenBank under the accession numbers MK778892–K778899 and MK778901–MK778907.

## Ethics Statement

Permission was obtained from animal owners or managers before collecting specimens, and no specific permits were required for the described field studies. All work involving animals was carried out in accordance with the Regulations for the Administration of Affairs Concerning Experimental Animals. The Research Ethics Committee of Henan Agricultural University reviewed and approved our study (approval no. LVRIAEC 2017-019).

## Author Contributions

MQ and L-XZ designed the study. YZ, Y-XJ, J-MX, D-YT, A-YZ, and BJ collected and analyzed the specimens. D-FL and Z-HC analyzed the data. D-FL, MQ, and L-XZ wrote the manuscript. All authors read and approved the final manuscript.

## Conflict of Interest

The authors declare that the research was conducted in the absence of any commercial or financial relationships that could be construed as a potential conflict of interest.

## Footnotes

1 http://www.clustal.org/

2 https://www.ncbi.nlm.nih.gov/

3 https://www.ncbi.nlm.nih.gov/genbank/

4 http://mrbayes.sourceforge.net/
